# *TFE3* Alleviates Hepatic Steatosis through Autophagy-Induced Lipophagy and *PGC1α*-Mediated Fatty Acid β-Oxidation

**DOI:** 10.3390/ijms17030387

**Published:** 2016-03-18

**Authors:** Jie Xiong, Kezhou Wang, Jiangping He, Guangya Zhang, Dandan Zhang, Fengling Chen

**Affiliations:** 1 Department of Endocrinology, Shanghai Ninth People’s Hospital, Shanghai Jiao Tong University School of Medicine, Shanghai 201999, China; jie_xiong@yeah.net (J.X.); jiangping_he@yeah.net (J.H.); zhang_guangya@126.com (G.Z.); zhang_daner@sina.com (D.Z.); 2 Department of Gastroenterology, Shanghai General Hospital, Shanghai Jiao Tong University School of Medicine, Shanghai 200080, China; 3 Department of Pathology and Pathophysiology, Dalian Medical University, Dalian 116044, China; wangkezhou123@sina.com

**Keywords:** TFE3, hepatic steatosis, autophagy, PGC1α, β-oxidation

## Abstract

Autophagy flux deficiency is closely related to the development of hepatic steatosis. Transcription factor E3 (*TFE3*) is reported to be a crucial gene that regulates autophagy flux and lysosome function. Therefore, we investigated the role of *TFE3* in a cell model of hepatic steatosis. We constructed L02 hepatocyte lines that stably over-expressed or knocked down the expression of *TFE3*. Subsequently, the effects of *TFE3* on hepatocellular lipid metabolism were determined by autophagy flux assay, lipid oil red O (ORO) staining, immunofluorescence staining, and mitochondrial β-oxidation assessment. Finally, we analyzed whether peroxisome proliferative activated receptor gamma coactivator 1α (*PGC1α*) was the potential target gene of *TFE3* in the regulation of hepatic steatosis using a chromatin immunoprecipitation (CHIP) assay and a luciferase reporter system. We found that overexpression of *TFE3* markedly alleviated hepatocellular steatosis. On the contrary, downregulation of *TFE3* resulted in an aggravated steatosis. The mechanistic studies revealed that the *TFE3*-manipulated regulatory effects on hepatocellular steatosis are dependent on autophagy-induced lipophagy and *PGC1α*-mediated fatty acid β-oxidation because blocking these pathways with an *Atg5* small interfering RNA (siRNA) or *PGC1α* siRNA dramatically blunted the *TFE3*-mediated regulation of steatosis. In conclusion, *TFE3* gene provides a novel insight into the treatment of hepatic steatosis and other metabolic disease.

## 1. Introduction

Non-alcoholic fatty liver disease (NAFLD) is a chronic liver disease with increasing incidence worldwide; it ranges from simple steatosis to steatohepatitis with progressive fibrosis and, ultimately, cirrhosis [1]. NAFLD is considered the hepatic event in an overall disturbed metabolic status and is, therefore, closely related to common metabolic syndrome risk factors such as obesity, insulin resistance, hyperlipidemia and hypertension. The main feature of NAFLD pathogenesis is the accumulation of triglyceride (TG) in the liver [2]. The imbalance between the synthesis and lipolysis of TG is a key pathogenic process in the development of NAFLD; thus, strategies that modulate the synthesis or lipolysis of TG may be useful therapeutic treatments to alleviate the progression of NAFLD [3].

Autophagy has been shown to play important roles in the pathophysiology of many diseases including NAFLD [4,5,6,7]. It can regulate the intracellular lipid level by breaking down lipid droplets, and this facet of autophagy has been termed lipophagy [8]. Mice with chronic obesity or insulin resistance, which are prone to NAFLD, show notably decreased expression of hepatic autophagy markers. The insufficient fusion between autophagosomes and lysosomes or lysosomal dysfunction may explain the inhibitory effect of a high fat diet (HFD) on autophagy, resulting in lipid accumulation [9,10,11]. Hepatocyte-specific autophagy-deficient mice (*Atg7* knockout) exhibit increased intracellular TG accumulation when fed an HFD, which is due to impaired lipolysis and a subsequent reduction in fatty acid β-oxidation but not to increased TG synthesis. In contrast, autophagy induction via liver-specific over-expression of *Atg7* or pharmacological agents such as rapamycin and carbamazepine improves the metabolic state and reduces steatosis [12,13,14]. These findings further demonstrate a lipolytic function of autophagy; thus, therapeutic strategies aimed at increasing autophagic functions may provide an attractive approach to prevent NAFLD and its associated pathologies.

Transcription factor E3 (*TFE3*) is a member of the basic helix-loop-helix leucine zipper family of transcription factors. It recognizes a 10-base pair motif (GTCACGTGAC) known as the coordinated lysosomal expression and regulation (CLEAR) element that is enriched in the promoters of numerous autophagy-lysosomal pathway-related genes. *TFE3* directly binds to the CLEAR elements present in the promoter region of these autophagy-lysosomal pathway-related genes; thus, over-expression of *TFE3* induces a significant increase in the number of lysosomes and autophagy flux [15,16]. Given that NAFLD is characterized by an impairment of autophagy-mediated lipolysis and lysosome function, we speculated that TFE-mediated modifications of lysosome biogenesis and autophagy flux could alleviate the TG accumulation in NAFLD.

In the present study, we show that over-expression of *TFE3* ameliorates the steatosis in hepatocytes exposed to free fatty acids (FFAs). Oppositely, knockdown of *TFE3* aggravates this pathlogical process. Furthermore, we demonstrate that the effects of TFE3 on hepatic steatosis dependent on the autophagy-induced lipophagy and *PGC1α*-mediated fatty acid β-oxidation.

## 2. Results and Discussion

### 2.1. Autophagy Flux Is Impaired in Free Fatty Acids (FFAs) Induced Hepatocellular Steatosis and Transcription Factor E3 (TFE3) May Be Involved in Dysfunctional Hepatic Lipid Metabolism

**Figure 1:**
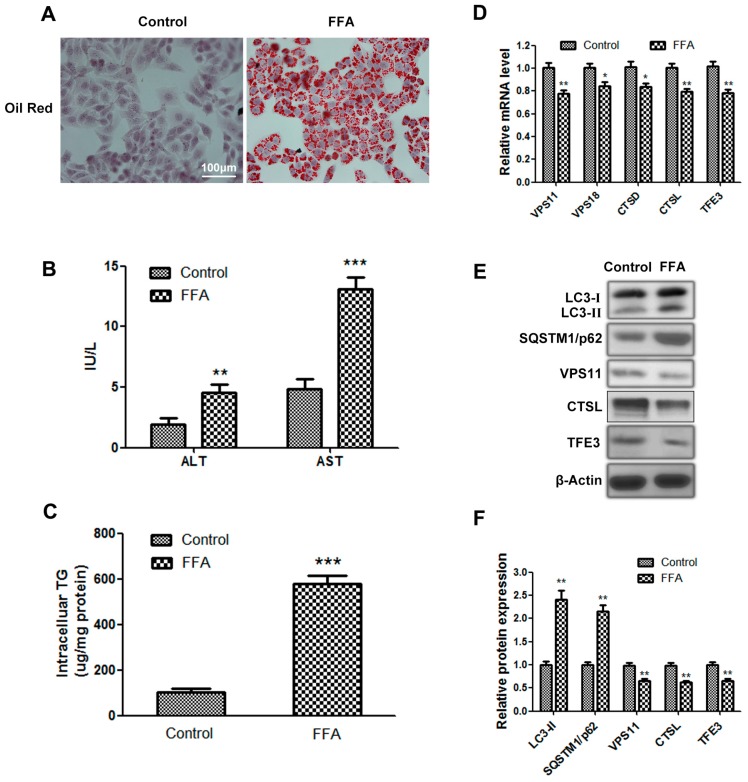
Autophagy flux is impaired in free fatty acid (FFA)-induced hepatocellular steatosis, and transcription factor E3 (*TFE3*) may be involved in this process. (**A**) Representative image of lipid oil red O (ORO)-stained cells, which were exposed to 1 mM FFA mixture for 24 h, compared with cells to which no FFA was added. scale bar, 100 μm; (**B**) alanine aminotransferase (ALT) and aspartate aminotransferase (AST) levels in cell supernatant; (**C**) intracellular TG contents; (**D**) qPCR data showing mRNA levels of different genes regulating fusion between autophagosomes and lysosomes (*VPS11*, *VPS18*), lysosome hydrolysis capacity (*CTSD*, *CTSL*) and *TFE3*; (**E**,**F**) Immunoblotting and densitometric analysis of autophagy flux related proteins (*LC3*, *SQSTM1/p62*), *VPS11*, *CTSL*, *TFE3* and internal control protein *β-Actin*. Representative images are shown, and data are presented as the means ± SEM of three independent experiments. * *p* < 0.05, ** *p* < 0.01, and *** *p* < 0.001 *versus* the control group.

First, we established a cell model of hepatic steatosis as mentioned in experimental section. The FFA group showed increased lipid accumulation (Figure 1A,C) and elevated aminotransferase levels, which are generally accompanied by hepatic steatosis (Figure 1B). These results indicated that the *in vitro* steatosis model, which exhibited intracellular TG accumulation, had been successfully established. The protein level of the autophagosome marker *LC3-II* in the FFA group was higher than the control group. Nevertheless, the expression of *SQSTM1/p62*, which is a selective substrate of autophagy and specifically degraded by autophagy, was also increased in the FFA group. Based on previous reports, impaired fusion of autophagosomes and lysosomes or lysosome dysfunction may account for this aberrant result. We found that the mRNA and protein levels of vacuolar protein sorting 11 (*VPS11*) and *VPS18*, which are mainly localized to the lysosomal membrane and play important roles in mediating the fusion between autophagosomes and lysosomes, were reduced in the FFA group. Meanwhile, we also observed decreased mRNA and protein levels of cathepsin D (*CTSD*) and cathepsin L (*CTSL*), which represent the lysosome hydrolysis capacity in the FFA group (Figure 1D–F). These data suggest that autophagy flux is impaired in FFA-induced hepatocellular steatosis due to lysosomal dysfunction. Given that *TFE3* is certified to be a crucial gene in regulating the autophagy-lysosomal pathway, we analyzed the expression of *TFE3* in this model of hepatic steatosis. The mRNA and protein levels of *TFE3* were decreased in the FFA group (Figure 1D–F); thus, we speculated that TFE3 may be involved in hepatic steatosis.

### 2.2. Overexpression of TFE3 Augments Autophagy Flux, and Knockdown of TFE3 Produces the Opposite Results

**Figure 2:**
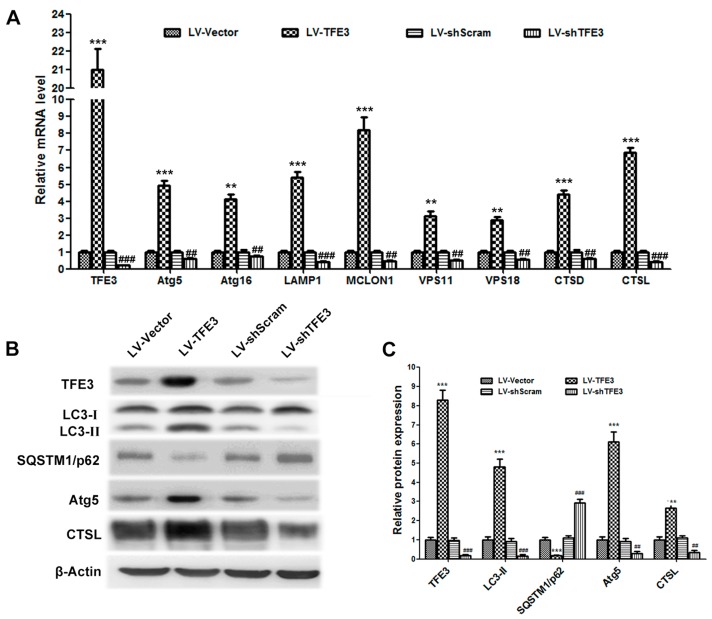

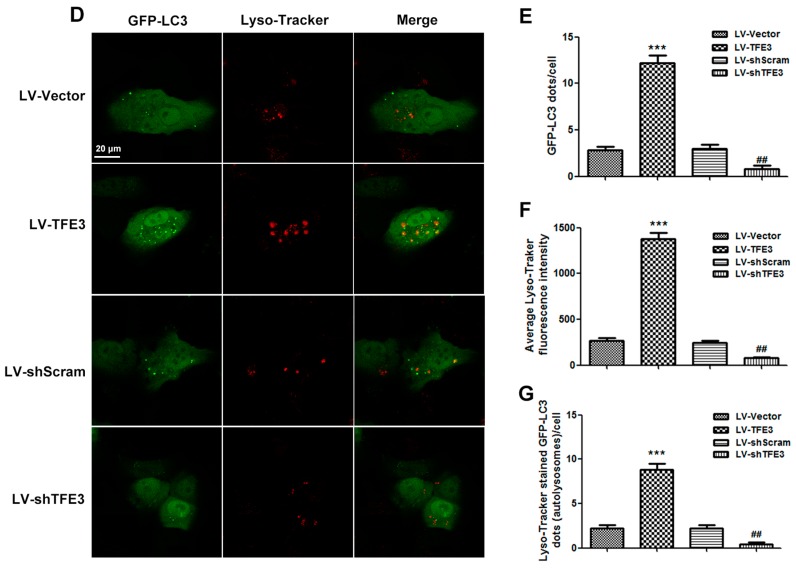
Overexpression of *TFE3* augments autophagy flux, and knockdown of *TFE3* produces opposite results. (**A**) The overexpression or knockdown of *TFE3* was initiated by adding doxycycline to the culture medium. The cells were exposed to 1 mM FFA mixture for 24 h, and then cells were cultured in medium without FFA for an additional 24 h. The mRNA levels of *TFE3* and representative genes regulating autophagosomes (*Atg5*, *Atg16*), lysosomal membrane proteins (*LAMP1*, *MCOLN1*), the fusion between autophagosomes and lysosomes (*VPS11*, *VPS18*), and lysosomal hydrolases (*CTSD*, *CTSL*) are shown; (**B**,**C**) Immunoblotting and densitometric analyses of *TFE3*, *LC3*, *SQSTM1/p62*, *Atg5*, *CTSL* and *β-Actin*; (**D**) The pEGFP-*LC3* plasmid was transfected into L02 cells 12 h before they were exposed to the same treatments described in (**A**), and then autophagic flux was analyzed by observing the GFP-*LC3* dots and LysoTracker staining. Scale bar, 20 μm. Representative images of three independent experiments are shown; (**E**) Quantification of the GFP-*LC3* dots in each cell. Fifteen cells were counted, and the data are presented as means ± SEM; (**F**) the lysosomes were loaded with LysoTracker Red and visualized by confocal microscopy. The average LysoTracker Red fluorescence was expressed as the mean fluorescence intensity; (**G**) Quantification of the LysoTracker Red-stained GFP-*LC3* dots, which represent the number of autolysosomes per cell. ** *p* < 0.01, and *** *p* < 0.001 *versus* the LV-Vector group; ^##^ *p* < 0.01, and ^###^ *p* < 0.001 *versus* the LV-shScram group.

To evaluate the role of *TFE3* in hepatic steatosis, we generated recombinant lentiviruses (LV) expressing *TFE3* and a *TFE3*-specific shRNA to construct cell lines that overexpress *TFE3* or knockdown *TFE3* expression. *TFE3* is reported to be a master regulator of the expression of autophagic and lysosomal genes; thus, we detected the expression of autophagy-lysosomal pathway-related genes by qPCR and immunoblotting. Overexpression of *TFE3* (LV-*TFE3* group) in L02 cells increased the mRNA and protein levels of numerous genes, including those involved in the formation of autophagosomes (*Atg5* and *Atg16*), lysosomal transmembrane proteins (lysosomal-associated membrane protein 1(*LAMP1*) and mucolipin 1 (*MCOLN1*)), lysosomal hydrolases (*CTSD* and *CTSL*), and fusion proteins (*VPS11* and *VPS18*) (Figure 2A,B). The increased expression of *LC3-II* and the degradation of *SQSTM1/p62* confirmed that autophagy flux was induced by the overexpression of *TFE3*. However, *TFE3* knockdown (LV-sh*TFE3* group) resulted in reduced autophagy flux (Figure 2B,C). After the cells were transfected with the GFP-*LC3* plasmid to detect intracellular autophagosome formation, we found that the LV-*TFE3* group notably increased the number of GFP-*LC3* dots compared with the vector group, which implied that more autophagosomes had formed (Figure 2D,E). Lyso-Tracker Red, a fluorescent lysosome probe, was used to assess lysosomal activity. Increased Lyso-Tracker fluorescence intensity, which indicated elevated numbers and function of lysosomes, was observed in the LV-sh*TFE3* group compared with the vector group (Figure 2F). The Lyso-Tracker-stained GFP-*LC3* dots represented the fusion between autophagosomes and lysosomes, known as autolysosomes. We detected a dramatic increase in the number of autolysosomes in the LV-*TFE3* group compared with the LV-Vector group (Figure 2G). Accordingly, the LV-sh*TFE3* group produced opposite results compared with LV-*TFE3* group. These data demonstrate that overexpression of *TFE3* augments autophagy flux in hepatocyte steatosis; on the contrary, knockdown of *TFE3* decreased autophagy flux.

### 2.3. TFE3 Alleviates Hepatocyte Steatosis in an Autophagy-Mediated Lipophagy Dependent Way

**Figure 3:**
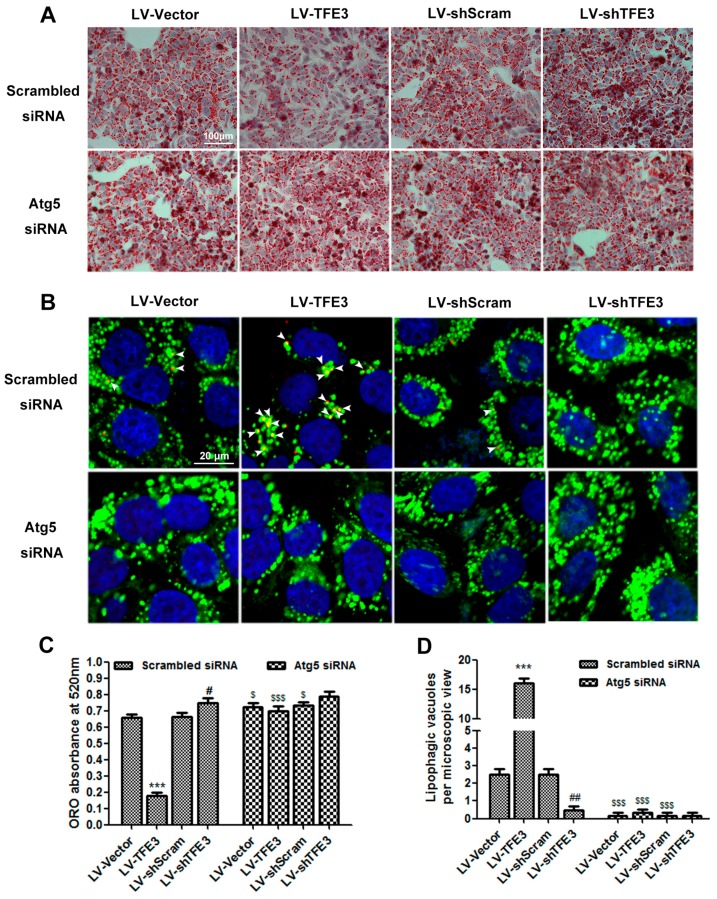

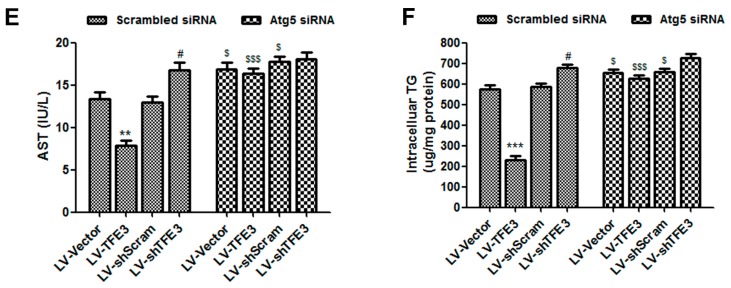
*TFE3* affects hepatocyte steatosis in an autophagy-mediated lipophagy dependent way. Cells were transfected with *Atg5* siRNA or scrambed siRNA; 12 h after transfection, the overexpression or knockdown of *TFE3* was initiated by adding doxycycline to the culture medium Cells were then exposed to 1 mM FFA mixture for 24 h and cultured in medium without FFA for an additional 36 h (**A**) or 24 h (**B**); (**A**) Lipid oil red O staining. Scale bar, 100 μm; (**B**) LC3 Immunofluorescence (red) and fluorescence staining of lipids with BODIPY 493/503 (green). As indicated by white arrowhead, the co-localization of *LC3* dots with the BODPY493/503 dye represents the induction of lipophagy (yellow). Scale bar, 20 μm; (**C**) Quantify ORO by measuring the absorbance at 520 nm; (**D**) quantification of averaged lipophagic dots in five microscopic fields; (**E**) AST level in cell supernatant of each group; (**F**) Intracellular TG content of each group. Representative images are shown, and data are presented as the means ± SEM of three independent experiments. ** *p* < 0.01, and *** *p* < 0.001 *versus* the LV-Vector group; ^#^ *p* < 0.05, and ^##^ *p* < 0.01 *versus* the LV-shScram group; ^$^ *p* < 0.05, and ^$$$^ *p* < 0.001 *versus* the corresponding group that transfected with scrambled siRNA.

To determine the role of *TFE3*-induced autophagy in FFA-induced hepatocyte steatosis, we used an *Atg5* siRNA to block autophagosome formation and measured its effect on steatosis by lipid oil red O (ORO) staining (absorbance measured at 520 nm) and determining the aminotransferase and TG contents. The FFA-induced hepatocyte steatosis was significantly alleviated in the LV-*TFE3* group and aggravated in the LV-sh*TFE3* group. Inhibition of autophagy by the *Atg5* siRNA nearly abolished the improvements in steatosis in the LV-*TFE3* group (Figure 3A). In addition, we also observed aggravated steatosis in the vector group when the cells were co-transfected with *Atg5* siRNA. These observations suggest that *TFE3*-mediated autophagy is involved in FFA-induced steatosis. However, we did not observe a significant difference in the LV-sh*TFE3* + *Atg5* siRNA group compared with the LV-sh*TFE3* + Scrambled siRNA group. This may have occurred because the autophagy flux in the LV-sh*TFE3* group was already significantly decreased; therefore, the application of the *Atg5* siRNA made almost no difference on autophagy between these two groups (Figure 3C,E,F). Furthermore, co-localization of the *LC3* dots (red) with the lipophilic dye BODIPY493/503 (green), which indicates the induction of lipophagy (yellow), was obviously visible in the LV-TFE3 group and almost absent in the LV-sh*TFE3* group. In accordance with a functional role for autophagy-mediated lipophagy, blocking autophagy with *Atg5* siRNA significantly reduced the number of BODIPY493/503-stained dots in the LV-*TFE3* + *Atg5* siRNA group compared with the LV-*TFE3* + Scrambled siRNA group. Likewise, the utilization of *Atg5* siRNA also decreased the number of BODIPY493/503-stained dots in the vector group, and there was no obvious difference between the LV-sh*TFE3* + Scrambled siRNA group and the LV-sh*TFE3* + *Atg5* siRNA group (Figure 3B,D). These data suggest that *TFE3* alleviates hepatocyte steatosis in an autophagy-mediated lipophagy-dependent way.

### 2.4. TFE3 Alleviates Hepatocyte Steatosis by Increasing Peroxisome Proliferative Activated Receptor Gamma Coactivator 1α (PGC1α)-Dependent Mitochondrial Fatty Acid β-Oxidation

**Figure 4:**
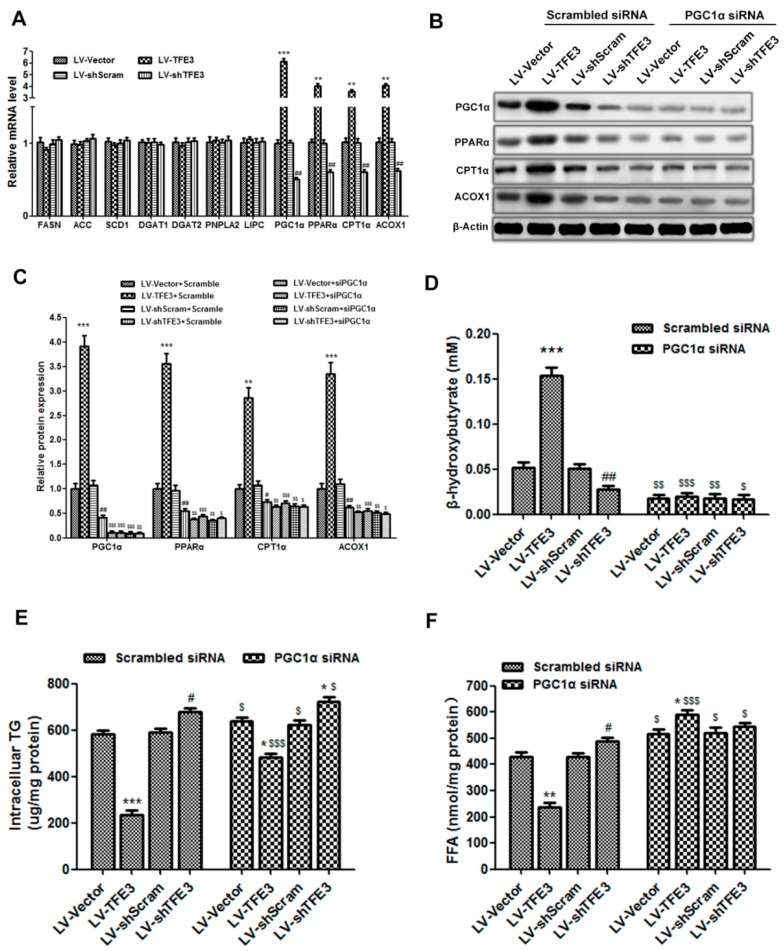
*TFE3* regulates hepatocyte steatosis through *PGC1α*-mediated mitochondrial fatty acid β-oxidation. (**A**) mRNA levels of genes regulating lipogenesis and TG synthesis (*FASN*, *ACC*, *SCD1*, *DGAT1*, *DGAT2*), the classical TG lipolytic enzymes (*PNPLA2*, *LIPC*), and those regulating mitochondrial β-oxidation (*PGC1α*, *PPARα*, *CPT1α*, *ACOX1*); (**B**,**C**) Immunoblotting and densitometric analyses of *PGC1α*, *PPARα*, *CPT1α*, *ACOX1* and *β-Actin*; (**D**) The amounts of β-hydroxybutyrate released in the medium were determined; (**E**) Intracellular TG content of each group; (**F**) FFAs level of each group. Representative images are shown, and the data are presented as the means ± SEM of three independent experiments. * *p* < 0.05, ** *p* < 0.01, and *** *p* < 0.001 *versus* the LV-Vector group; ^#^ *p* < 0.05, and ^##^ *p* < 0.01 *versus* the LV-shScram group; ^$^ *p* < 0.05, ^$$^ *p* < 0.01, and ^$$$^ *p* < 0.001 *versus* the corresponding group that was transfected with the scrambled siRNA.

The lipolysis of TG results in the production of FFAs and glycerol; thus, the increased autophagy-mediated lipophagy would generate excess levels of FFAs in hepatocytes. FFAs are considered to be toxic because they induce lipid peroxidation and lipoapoptosis. There are two metabolic pathways for these FFAs: one is re-esterification back to TG for storage, and the other is oxidation, generally in the mitochondria, to supply energy for physiological processes. Taking into account that these two pathways for FFAs may affect the TG contents in hepatocytes, we analyzed the expression of some of the key genes involved in these pathways. We did not observe significant differences in the mRNA levels of genes related to lipogenesis and TG synthesis: fatty acid synthase (*FASN*), acetyl-CoA carboxylase (*ACC*), stearoyl-CoA desaturase 1 (*SCD1*), diacylglycerol *O*-acyltransferase 1 (*DGAT1*), and *DGAT2*. In addition, no obvious changes were detected in the mRNA levels of the genes in the classical lipolysis pathway: patatin-like phospholipase domain containing 2 (*PNPLA2*) and lipase C (*LIPC*), further indicating that *TFE3*-mediated lipophagy had a functional role in the catabolism of TG. Nevertheless, the expressions of genes that modulate FFA β-oxidation: *PGC1α*, peroxisome proliferator activated receptor α (*PPARα*), carnitine palmitoyltransferase 1α (*CPT1α*), and acyl-CoA oxidase 1 (*ACOX1*), were dramatically increased in the LV-TFE3 group; conversely, their expression levels were notably decreased in the LV-sh*TFE3* group (Figure 4A).

Considering that *PGC1α* is known to be a pivotal regulator of mitochondrial β-oxidation and liver lipid metabolism, we used siRNA to knockdown the expression of *PGC1α* and observe its effects on *TFE3*-regulated β-oxidation. The TFE3-induced expression of PGC1α and β-oxidation related genes was noticeably abolished in the LV-*TFE3* + *PGC1α* siRNA group compared with the LV-*TFE3* + Scrambled siRNA group. It was worth noting that the expression of *PGC1α* and β-oxidation related proteins was also decreased to some extent in the LV-sh*TFE3* + Scrambled siRNA group compared with the vector control group. As expected, the levels of these proteins were more substantially decreased in the LV-sh*TFE3* + *PGC1α* siRNA group compared with the LV-sh*TFE3* + Scrambled siRNA group, probably due to a further *PGC1α* deficiency. The β-oxidation related proteins decreased to approximate levels in all of the four groups when *PGC1α* siRNA were added (Figure 4B,C). These results prompted us to speculate that *PGC1α* may be a target gene of TFE3 in regulating β-oxidation. The *PGC1α*-dependent enhancement of β-oxidation by *TFE3* was further shown by measuring the level of β-hydroxybutyrate, a metabolic product of β-oxidation. The LV-*TFE3* group displayed significant high level of β-hydroxybutyrate compared with the LV-Vector group, indicating the reinforcement of β-oxidation; whereas, the LV-sh*TFE3* group produced the opposite result. The level of β-hydroxybutyrate in the LV-*TFE3* group was markedly declined when *PGC1α* siRNA was added and had almost no difference compared with the control group co-transfected with *PGC1α* siRNA. The other groups all showed a decreased level of β-hydroxybutyrate when co-transfected with *PGC1α* siRNA compared with the corresponding group transfected with scrambled siRNA (Figure 4D). These data demonstrated that the regulatory effect of *TFE3* on β-oxidation was dependent on *PGC1α*. Regarding hepatocyte steatosis, we observed that the *TFE3-*mediated regulatory effects on the intracellular TG content were more or less reversed when co-transfected with *PGC1α* siRNA. The most reasonable interpretation for these results is that the FFAs were re-esterified to TG, due to the lack of *PGC1α*-mediated β-oxidation. Even so, the TG content of the LV-*TFE3* + *PGC1α* siRNA group was yet lower than the LV-Vector + *PGC1α* siRNA group. This may occurred because the generated FFAs by lipophagy were too excessive and the capacity of re-esterification was limited. It should be mentioned that the TG content in the LV-sh*TFE3* group was still higher than the LV-shScram group even though co-transfected with *PGC1α* siRNA. This result indicated that *PGC1α* may only play a crucial role in the subsequent FFAs β-oxidation rather than the *TFE3*-mediated lipophagy (Figure 4E). Moreover, we also detected the FFAs level in each group. As expected, the LV-*TFE3* group showed decreased level of FFAs and the LV-sh*TFE3* group resulted in an elevated level, due to the regulatory effect of *TFE3* on β-oxidation. When *PGC1α* siRNA was added to block FFAs β-oxidation, all the groups showed an increased level of FFAs but especially obvious in the LV-*TFE3* + *PGC1α* siRNA group. The excessive production of FFAs by *TFE3-*mediated lipophagy together with the absence of subsequent β-oxidation induced by *PGC1α* siRNA may account for this result (Figure 4F).

**Figure 5:**
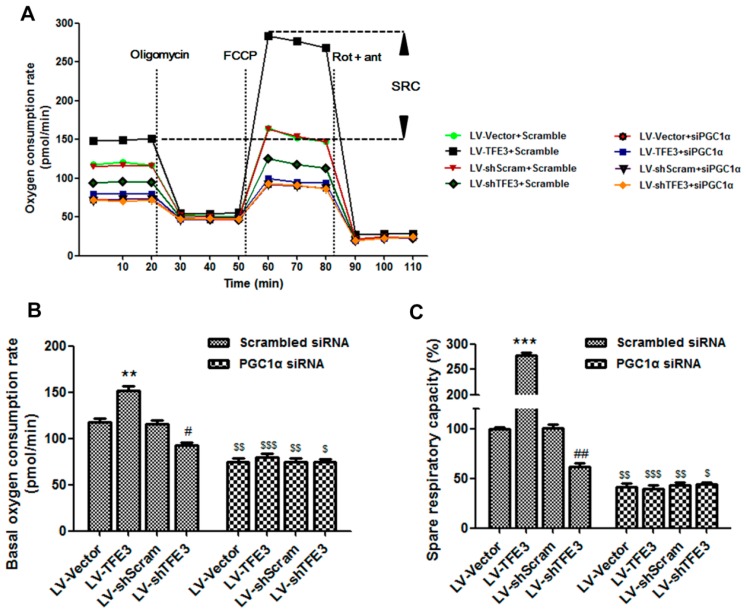
*PGC1α*-mediated mitochondrial fatty acid β-oxidation is measured by the oxygen consumption rate (OCR) and spare respiratory capacity (SRC). (**A**) The OCR was measured continuously throughout the experimental period, at baseline, and in the presence of the indicated drugs: oligomycin (1 μM); carbonyl cyanide-4-trifluoro methoxy phenyl hydrazone (FCCP, 1 μM); rotenone (1 μM) plus antimycin A (1 μM). A representative plot shows the *PGC1α*-dependent *TFE3*-induced increase in the spare respiratory capacity; (**B**) Initial basal OCR of each group (bottom horizontal dashed line); (**C**) The SRC of each group was quantitated by calculating the difference between the maximal uncontrolled OCR (top horizontal dashed line) and the initial basal OCR (bottom horizontal dashed line). Representative results from three independent experiments are shown, and the data are presented as the means ± SEM of three technical replicates. ** *p* < 0.01, and *** *p* < 0.001 *versus* the LV-Vector group; ^#^ *p* < 0.05, and ^##^ *p* < 0.01 *versus* the LV-shScram group; ^$^ *p* < 0.05, ^$$^ *p* < 0.01, and ^$$$^ *p* < 0.001 *versus* the corresponding group that transfected with scrambled siRNA.

Because FFA β-oxidation is associated with mitochondrial electron and oxygen consumption, we performed a seahorse metabolic analysis to evaluate the respiratory capacity. Cells that overexpressed *TFE3* displayed an enhanced mitochondrial oxygen consumption rate (OCR) and markedly increased spare respiratory capacity (SRC), indicating an increased commitment to oxidative phosphorylation. In contrast, *TFE3* knockdown cells exhibited reduced OCR and SRC, which further confirmed that *TFE3* modulates mitochondrial function. Interestingly, these effects were blunted in the presence of *PGC1α* siRNA, which was particularly apparent in the LV-*TFE3* group (Figure 5A–C). Overall, these data suggest that *TFE3* alleviates hepatocyte steatosis by increasing *PGC1α*-mediated mitochondrial fatty acid β-oxidation.

### 2.5. TFE3 Regulates PGC1α via Binding to Its Promoter Region

**Figure 6:**
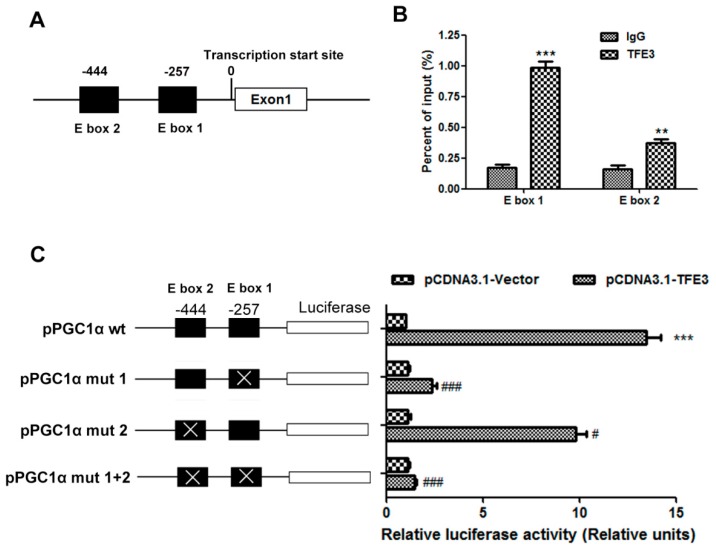
*TFE3* regulates *PGC1α* via binding to its promoter region. (**A**) Structure of the *PGC1α* promoter displayed the location of E-boxes; (**B**) The binding between *TFE3* and *PGC1α* promoter was performed by CHIP analysis. Soluble chromatin was immunoprecipitated with anti-TFE3 and IgG antibody. The immunoprecipitates were analyzed by qPCR using primers flanking the E-boxes sequences in the *PGC1α* promoter (denominated here as E-box1 and E-box 2). The value is shown as the percentage relative to the input. (**C**) Mutations of the E-boxes attenuated the luciferase activity. The *PGC1α* promoter (*PGC1α* wt), −444 site mutant promoter (*PGC1α* mut 1), −257 site mutant promoter (*PGC1α* mut 2), and promoter in which both the −444 and −257 sites were mutated (*PGC1α* mut all) were cloned into the pGL3-luciferase vector. The luciferase activity was measured after the cells were co-transfected with *TFE3* and the wt or mutated *PGC1α* promoter constructs. A control Renilla plasmid was co-transfected for normalization purposes. The luciferase activity in cells that were co-transfected with the empty vector plasmid was set as 1, and the fold change was calculated relative to this level of activity. Representative results from three independent experiments are shown, and the data are presented as the means ± SEM of three technical replicates. ** *p* < 0.01, and *** *p* < 0.001 *versus* the IgG control group (**B**) or pCDNA3.1-Vector group (**C**); ^#^ *p* < 0.05, and ^###^ *p* < 0.001 *versus* the corresponding group that transfected with pPGC1α wt.

We speculated that *TFE3* may directly regulate the expression of *PGC1α* by binding to and transactivating its promoter region. It is well established that transcription factors, belonging to the basic helix-loop-helix leucine zipper family, specifically bind to the E-box (CANNTG) response elements present in the promoter region of their downstream target genes. Thus, two putative *TFE3* binding sites, which contain the E-box sequences, were identified in the *PGC1α* promoter region at −444 and −257 bp upstream of the transcription start site (Figure 6A). Chromatin immunoprecipitation (CHIP) assays were performed to investigate whether endogenous *TFE3* protein could be recruited to these two putative binding sites. We found that *TFE3* could bind to both of these E-box sites but predominantly bound the E-box 1 (−257 bp) sequence (Figure 6B). A luciferase reporter system was constructed to evaluate whether *TFE3* could transactivate the *PGC1α* promoter region. The upstream regulatory promoter region of the human *PGC1α* gene containing the two E-boxes was cloned into a luciferase reporter. The *PGC1α* luciferase reporter plasmid was co-transfected into L02 cells with the pCDNA3.1-*TFE3* plasmid. As a result, co-transfection with pCDNA3.1-*TFE3* notably increased the luciferase activity of PGC1α by approximately 12-fold. Then, site-specific mutation (*PGC1α* mut 1, *PGC1α* mut 2 and *PGC1α* mut 1 + 2) was generated to evaluate the importance of the two E-box sequences respectively. The mutation of each E-box attenuated the luciferase activity of the *PGC1α* promoter, with the greater inhibition occurring for the E-box 1 mutation, and the luciferase activity was almost completely abrogated in the promoter in which both sites were mutated (Figure 6C). These data suggest that *TFE3* transcriptionally regulates *PGC1α* expression by binding to E-box sequences in the *PGC1α* promoter region.

Autophagy is derived from the Greek terms “auto” and “phagos” and literally means “self-eating”. Basal autophagy serves as a housekeeper in the continuous turnover of cellular contents, thereby removing damaged or dysfunctional cellular contents and supplying substrates for energy metabolism. Three types of autophagy have been identified: macroautophagy, chaperone-mediated autophagy, and microautophagy. Among the three types of autophagy, macroautophagy (hereafter called autophagy) is considered to play the most important role in pathophysiology and has been well studied in recent years [17]. In autophagy, the cytoplasmic material is sequestrated in a double membrane structure called the autophagosome, which fuses with a lysosome to form an autolysosome where its contents will be degraded. The process of autophagosome formation involves three major steps, namely initiation, nucleation, and elongation/enclosure, which require numerous different autophagy-related proteins, such as the *Atg* proteins. Two conjugation systems are involved in the elongation/enclosure step. The first is the formation of the *Atg12-Atg5-Atg16* complex, and the second involves the cleavage of *LC3/Atg8* by *Atg4*, leading to the formation of the soluble LC3-I protein, which is then conjugated to phosphatidylethanolamine via the activities of *Atg7* and *Atg3* [7]. This lipid conjugation forms the autophagic double-membrane-associated *LC3-II* protein, allowing the closure of the autophagic vacuole. Thus, *LC3-II* is frequently used as a marker for autophagy [4,18].

Emerging evidence indicate that autophagy and lipid metabolism are correlated. Therefore, impaired autophagy may contribute to the pathogenesis of NAFLD [19]. Singh *et al.* were the first to convincingly correlate autophagy with lipid metabolism and they named this novel selective pathway in lipid breakdown “lipophagy” [8]. In the present study, we observed decreased autophagy flux and lysosome dysfunction in the hepatocytes that were stimulated with exogenous FFAs, and these results are consistent with previous research [11,20]. Based on reports showing that increased autophagy decreased TG accumulation in NAFLD and that *TFE3* plays a key role in autophagy flux and lysosome biogenesis [21,22], we thus investigated the function of *TFE3* in TG accumulation. In our study, we showed that the *TFE3* expression levels were decreased in hepatocytes that were exposed to exogenous FFAs. These data implied that the TFE3 expression level may be closely associated with the metabolic functions of the liver. Thus, we performed genetic *TFE3* over-expression or knockdown experiments to elucidate the details of the potential mechanism. We observed a dramatic increase in autophagy flux and the number of lysosomes in the over-expression group and a corresponding decrease in the knockdown group. These results were in accordance with a previous study of the effects of *TFE3* [16]. *TFE3* overexpression protected hepatocytes from steatosis; conversely, *TFE3* knockdown aggravated lipid accumulation. To explore whether the attenuation of steatosis by TFE3 was autophagy-dependent, we inhibited the induction of autophagy with siRNA directed against *Atg5*, which is a crucial gene involved in autophagosome formation. We demonstrated that the autophagy-lysosomal pathway is essential in reducing the *TFE3*-induced intracellular lipid content. This result was further corroborated by immunofluorescence and showed that the lipids were co-localized with the autophagy marker *LC3*, indicating the occurrence of lipophagy. Theoretically, an alleviation of the intracellular TG accumulation may be caused by both decreased TG synthesis and enhanced lipolysis. We did not observe significant changes in the expression of genes involved in TG synthesis or the classical lipolysis pathway. These findings further supported the hypothesis that the protective effect of *TFE3* on hepatocyte steatosis was dependent on autophagy-lysosomal pathway-mediated lipophagy.

The reinforcement of lipophagy in hepatocytes produces increased levels of FFAs, which were proven to be cytotoxic. The excess FFA levels in hepatocytes are closely related to lipid peroxidation, the production of inflammatory cytokines, and cell apoptosis; therefore, the utilization or metabolism of FFAs appears to be particularly important [23,24]. Normally, FFAs in hepatocytes may follow two principal pathways: one consisting of β-oxidation and the other consisting of storage as TG that is controlled by the rate-limiting enzyme *DGAT*. The β-oxidation primarily occurs within mitochondria and progressively shortens FFAs into acetyl-CoA subunits, which either condense into ketone bodies to serve as oxidizable energy substrates or enter into the tricarboxylic acid cycle for further oxidation to water, carbon dioxide and ATP [1,2]. Extensive experimental observations indicated that *PPARα* activation prevents hepatic TG infiltration by increasing the rate of FFA catabolism [3,25]. Although it is named *PPARγ* coactivator, *PGC1α* also acts as a coactivator of *PPARα* in the transcriptional control of mitochondrial fatty acid β-oxidation. Thus, the *PGC1α-PPARα* complex plays a key role in the transcriptional control of genes encoding proteins involved in mitochondrial fatty acid β-oxidation [26]. Our study showed that *TFE3* overexpression increased the expression of *PGC1α* and β-oxidation-related proteins. The enhanced β-oxidation was further verified by increased ketogenesis and spare respiratory activity. Simultaneously, we demonstrated that the role of *TFE3* in reinforcing mitochondrial β-oxidation relied on the elevated expression of *PGC1α* because knockdown of *PGC1α* with siRNA nearly abolished this effect. It is noteworthy that hepatic steatosis generally exerts mitochondrial dysfunction due to the oxidative damage to the electron transport chain complexes and mitochondrial DNA (mtDNA), thus stimulation of mitochondrial biogenesis is also a strategy for the therapy of hepatic steatosis [27]. Currently, *PGC1α* is considered to be a pivotal regulator of mitochondrial biogenesis [28,29]; therefore, the TFE3-induced enhancement of β-oxidation may also couple with increased biogenesis of new fully functional mitochondria.

TFE3 had been reported to mediate metabolism by regulating the genes that directly participate in the insulin-signaling pathway [30,31]. However, the role of *TFE3* in TG metabolism had not been clearly elucidated. In the present study, we showed that TFE3 promoted TG lipolysis through an autophagy-lysosome pathway. *TFE3* was previously confirmed to regulate genes belonging to the E-box network [32]. Here, we identified two putative E-box sites in the promoter region of the *PGC1α* gene and demonstrated that TFE3 directly binds to these E-box sites by CHIP, PCR, and luciferase assays. Our results were consistent with previous reports showing that rapamycin or other autophagy-inducing agents could alleviate hepatic steatosis by stimulating both autophagy and fatty acid oxidation [12,13,33]. However, the lack of specificity, the effective dose and the absence of organ or cell selectivity are the major limitations of these compounds for clinical application. In the present study, by genetical gain and loss analyses, we showed that *TFE3* played a crucial role in autophagy-mediated lipophagy and the subsequent β-oxidation during hepatic TG metabolism. There are some limitations in our study, which should be addressed by further research. We only explore the role of *TFE3* in a model of hepatocellular steatosis *in vitro*, whether these results are consistent with *in vivo* study remains undefined. Hence, additional experiments with genetically engineered animal models such as liver-specific *TFE3*-overexpression or knockout mice should be conducted in the future to provide a more definitive mechanism of TFE3.

## 3. Materials and Methods

### 3.1. Reagents and Antibodies

Fetal bovine serum (FBS), culture media, TRIzol reagent, LysoTracker Red, BODIPY493/503, and the Lipofectamine 3000 transfection reagent were obtained from Invitrogen (Carlsbad, CA, USA). Palmitate (PA) and oleic acid (OA) were purchased from Sigma-Aldrich (St. Louis, MO, USA). Antibodies against *LC3*, *SQSTM1/p62*, and *TFE3* were obtained from Sigma-Aldrich. Antibodies against Cathepsin L, *PGC1α*, *PPARα*, *CPT1α*, and *ACOX1* were from Cell Signaling Technology (Danvers, MA, USA). Antibodies against *VPS11* and *β-Actin* were purchased from Santa Cruz Biotechnology (Santa Cruz, CA, USA). Unless otherwise specified, all other reagents were purchased from Sigma-Aldrich.

### 3.2. Cell Culture and FFA Treatment

The L02 human hepatocyte cell line was obtained from the China Cell Culture Center (Shanghai, China). The cells were cultured in Dulbecco’s Modified Eagle’s Medium (DMEM) supplemented with 10% FBS, penicillin G (100 U/mL), streptomycin (100 mg/mL), and l-glutamine (2 mM) at 37 °C in a humidified atmosphere of 5% CO_2_. After reaching 70% confluence, the cells were exposed to a 1 mM FFA mixture for 24 h to induce hepatocyte steatosis, as previously described [25]. Briefly, stock solutions of 50 mM oleate and 50 mM palmitate were prepared in culture medium containing 1% fatty acid-free bovine serum albumin. The 1 mM FFA mixture containing a 2:1 ratio of oleate/palmitate was diluted in culture medium to reach the desired final concentrations. The control cells were treated with the corresponding concentration of bovine serum albumin.

### 3.3. Construction of Inducible TFE3 Expression Cell Lines

The full-length human *TFE3* gene was amplified by polymerase chain reaction from the mRNA from HEK293 cells using the following primer pair: 5′-CGGGATCCATGTCTCATGCGGCCGAACCAG-3′ as the forward primer and 5′-ATGCGGCCGCTCAGGACTCCTCTTCCATGCTGAAGC-3′ as the reverse primer. Then, it was cloned into the doxycycline (Dox)-inducible lentiviral expression vector pLVX-Tight-Puro (Clontech, CA, USA) through the BamHI and NotI enzyme sites. For RNA interference, the DNA sequences corresponding to the short hairpin RNA (shRNA) sequences 5′-CCGGCAATGATGAAATG CTCAGCTACTCGAGTAGCTGAGCATTTCATCATTGTTTTT-3′ (top) and 5′-AATTAAAAACAATGATGAAATGCTCAGCTACTCGAGTAGCTGAGCATTTCATCATTG-3′ (bottom) against *TFE3* were cloned into the Dox-inducible lentiviral vector pLKO-Tet-On (Addgene, MA, USA). As a negative control, a recombinant lentiviral vector expressing a scrambled shRNA (shScram) was generated. We generated the lentivirus particles and used them to infect the L02 cell line to construct the Dox-inducible *TFE3* expression systems according to the manufacturer’s instructions. In this study, based on our preliminary experiments, the L02 cells infected with lentivirus-TFE3 (LV-*TFE3* group) would overexpress *TFE3* in the presence of 500 ng/mL doxycycline within 48 h. On the contrary, cells infected with lentivirus-sh*TFE3* (LV-sh*TFE3* group) would induce the knockdown of *TFE3* in the presence of 100 ng/mL doxycycline compared with the lentivirus-shScram (LV-shScram) group.

### 3.4. Oil Red O Staining and Lipid Content Measurement

Hepatic lipid accumulation was detected by ORO staining as previously described. Briefly, L02 cells were cultured overnight at a density of 50,000 cells per well in a 12-well plate and then exposed to the indicated treatments. The cells were then fixed in 4% paraformaldehyde for 15 min, washed three times with PBS, stained with 0.5% ORO for 10 min at room temperature, and counterstained with hematoxylin before microscopic examination. To quantify the ORO content, isopropanol was added to each sample, and samples were shaken at room temperature for 10 min. The absorbance was measured at 520 nm on a monochromator microplate reader. The triglyceride (TG) contents of hepatocytes were measured with a TG quantification kit (ab65336; Abcam, Cambridge, MA, USA).

### 3.5. Measurement of Biochemical Parameters

The biochemical parameters, including aspartate aminotransferase (AST), alanine aminotransferase (ALT) and FFA levels, were measured by spectrophotometry using commercial assay kits from Nanjing Jiancheng Bioengineering Institute (Nanjing, China) according to the manufacturer’s instructions. β-Hydroxybutyrate contents were detected using the colorimetric assay kit from Abcam (ab83390) according to the manufacturer’s instructions. The cellular protein contents were determined with a Bicinchoninic Acid (BCA) Protein Assay Kit (Pierce Biotechnology, Rockford, USA).

### 3.6. Autophagic Flux Analysis

The pEGFP-*LC3* plasmid (a kind gift from Jinke Cheng, Shanghai Jiao Tong University School of Medicine) was transfected into L02 cells with the Lipofectamine 3000 transfection reagent. Twelve hours later, the overexpression or knockdown of *TFE3* was initiated by adding doxycycline to the culture medium. The cells were exposed to a 1 mM FFA mixture for 24 h, and then the cells were cultured in medium without FFA for an additional 24 h. Thereafter, the cells were incubated with 200 nM LysoTracker Red for 30 min at 37 °C, the medium was replaced with fresh medium, and the cells were immediately observed under an LSM710 Carl Zeiss confocal microscope (Carl Zeiss AG, Jena, Germany) to analyze the intensity of the GFP-*LC3* dots and LysoTracker fluorescence.

### 3.7. RNA Purification and Quantitative Polymerase Chain Reaction (qPCR) Analysis

Total RNA was isolated using Trizol Reagent and transcribed into the complementary DNA using the QuantiTect reverse transcription kit (Qiagen, Hilden, Germany). Gene expression was determined by qPCR using the FastStart SYBR Green master (ROX) (Roche, Basel, Swizerland), and mRNA levels were normalized to the *GAPDH* gene. The sequences of the qPCR primers are listed in Table 1, and all primers were synthesized by Sangon Biotech (Shanghai, China).

### 3.8. Western Blot Analysis

Whole-cell lysates were prepared with RIPA buffer (Pierce) containing the Halt™ Protease and Phosphatase Inhibitor Cocktail (Pierce Biotechnology) according to the manufacturer’s instructions. The total protein concentration was determined using the BCA protein assay kit. The protein samples were separated on 8%–15% SDS-PAGE gels and then transferred to PVDF membranes (Millipore, Darmstadt, Germany). The membranes were blocked with 5% (*w*/*v*) dry milk for 1 h at room temperature and then incubated with the following primary antibodies overnight at 4 °C: LC3, *SQSTM1/p62*, *TFE3*, *CTSL*, *VPS11*, *PGC1α*, *PPARα*, *CPT1α*, *ACOX1*, and *β-Actin*. The binding of all antibodies was detected using an enhanced chemiluminescence detection system (Animal Genetics, Truro, UK) according to the manufacturer’s instructions. The intensity of the immunoreactive bands was determined using a GS-710 calibrated imaging densitometer (Bio-Rad, CA, USA).

### 3.9. RNA Interference

Cells were transfected with siRNAs targeting *Atg5* (Sigma, SASI_Hs01_00173156), *PGC1α* (Sigma, SASI_Hs01_00063323), or a scrambled siRNA (Sigma, SIC001) using Lipofectamine 3000 according to the manufacturer’s instructions. The cells were incubated with a transfection mixture containing a final siRNA concentration of 100 pM for 12 h.

### 3.10. BODIPY 493/503 Staining and Immunofluorescence Assay

Cells were transfected with *Atg5* siRNA or scrambled siRNA; 12 h after transfection, the overexpression or knockdown of *TFE3* was initiated by adding doxycycline to the culture medium. The cells were exposed to a 1 mM FFA mixture for 24 h and then cultured in medium without FFA for an additional 24 h. The cells were washed twice with PBS and fixed with 4% paraformaldehyde for 20 min at room temperature. After fixation, cells were washed four times with PBS, followed by incubation with blocking buffer (1.5 g glycine, 3 g BSA and 2 mL 0.5% (*w*/*v*) saponin in 100 mL PBS) for 45 min. The cells were incubated with a rabbit anti-LC3 antibody (1:400 dilution in antibody diluent: 100 mg BSA and 2 mL 0.5% saponin in 100 mL PBS) overnight at 4 °C. The cells were washed four times (10 min each) with PBS, followed by incubation with the secondary antibody (1:200 dilution of AlexaFluor 594-conjugated goat anti-rabbit IgG in antibody diluent) and BODIPY 493/503 (1 mg/mL concentration) for 1 h at room temperature. Then, the cells were washed four times with PBS followed by 4′, 6-diamidino-2-phenylindole (DAPI) staining using the mounting solution. Images of the cells were obtained using a confocal microscope. The yellow dots were defined as lipophagic vacuoles, which were quantified in at least 5 microscopic fields using ImageJ software (Bethesda, MA, USA).

### 3.11. Seahorse XF-96 Metabolic Flux Analysis

Oxygen consumption was measured at 37 °C using an XF-96 extracellular analyzer (Seahorse Bioscience Inc., North Billerica, MA, USA). L02 cells (5000) were seeded in 96-well plates and transected with either a *PGC1α* or negative control siRNA. Twelve hours after transfection, the overexpression or knockdown of *TFE3* was initiated by adding doxycycline to the culture media. After 24 h, the cells were exposed to a 1 mM FFA mixture for an additional 24 h. The medium was replaced with unbuffered DMEM, and the cells were incubated at 37 °C in a no CO_2_ incubator for 1 h. All reagents used in this experiment were adjusted to pH 7.4 on the day of the assay. Each data point represents an average from 3 different wells.

### 3.12. Chromatin Immunoprecipitation (CHIP) Assay

The cells were cross-linked in 1% formaldehyde at 37 °C for 15 min. After stopping the cross-linking by glycine, cells were washed three times with PBS and collected in PBS containing phosphatase and protease inhibitors. After centrifugation, cell pellets were resuspended with lysis buffer (containing phosphatase and protease inhibitors) and sonicated at 4 °C to shear DNA to lengths between 200 and 500-bp. The sonicated lysates were diluted 10-fold with immunoprecipitation buffer and rotated with 50% slurry of Protein A/G at 4 °C for 1 h to reduce non-specific binding. After the beads were pelleted by brief centrifugation, the *TFE3* immunoprecipitation antibody and the control rabbit IgG (Santa Cruz) antibody were added to the supernatant fraction respectively and incubated overnight at 4 °C. The immune complexes were collected by rotating with 50% slurry of Protein A/G in Tris-EDTA (TE) buffer at 4 °C for 1 h. After gentle centrifugation, the beads were roated gradiently with salt wash buffers and finally washed with TE buffer. The immune complexes were eluted from the beads with 1% SDS in TE, and the DNA–protein cross-links were reversed by treatment with NaCl and heating the samples at 65 °C for 6 h. After adding proteinase K, the products were purified by a PCR purification kit (Qiagen) and detected by qPCR. The primer sequences used for the qPCR analysis were as follows: 5′-CATTTTTCCTCTTCCCGGGCTC-3′ and 5′-TCTCCGACCTCCTCGCCATAG-3′ for PGC1α-E-box 1; and 5′-CGGCGTGGTCTGATTTAGTGG-3′ and 5′- TGTGCGTCTGTTTGGGGAGCT-3′ for PGC1α-E-box 2.

### 3.13. Luciferase Reporter

The human *PGC1α* promoter (1000 bp) containing two E-boxes was amplified by PCR using human genomic DNA as a template and was cloned into the pGL3 basic vector (Promega, Madison, WI, USA). The site mutation of the E-boxes was performed using the Quick-Change Site-Directed Mutagenesis kit (Stratagene, SanDiego, CA, USA) according to the manufacturer’s instructions. The sequences of primers were as follows: 5′-CACCCATCCATC***GT***CC***A***GCCCGCGGCCTCAC-3′ for E-box 1 and 5′-GAGTCAGCGCGCC***GT***CG***A***GACGGCCCCGGCT-3′ for E-box 2. The mutated sites in the primers listed above are highlighed by underlined bold text. The cells were split and plated in 24-well plates at a density of 5 × 10^4^ cells/cm^2^. The cells in each well were co-transfected with different combinations of the vectors containing the reporter and *TFE3* cDNA. A total of 50 ng of pGL3-*PGC1α* wild type (wt) (or each mutated construct), 50 ng of empty pGL3, and 250 ng of pCDNA3.1-*TFE3* or pCDNA3.1-Vector were used. At 36 h after the transfections were performed, cells were washed and lysed with lysis buffer for luciferase assay (Promega). The supernatant of each group was assayed for luciferase activity using the Dual-Luciferase Reporter Assay System (Promega).

### 3.14. Statistical Analysis

Data are presented as means ± standard error of the mean (SEM) and were compared between or among groups by a two-tailed unpaired Student’s *t*-test or by a one-way analysis of variance (ANOVA) with a *post hoc* Bonferroni multiple comparison test. *p* < 0.05 was considered statistically significant.

## 4. Conclusions

In summary, our findings demonstrate that *TFE3* triggers the activation of the autophagy-lysosomal pathway, thus leading to the induction of lipophagy and the subsequent *PGC1α*-dependent β-oxidation of FFAs. As a result of these changes, *TFE3* attenuates FFA-induced intracellular steatosis in hepatocytes. Therefore, *TFE3* may provide a novel therapeutic strategy for the treatment of NAFLD and other metabolic diseases.

## Figures and Tables

**Table 1:** qPCR Primers used in this work. All of the primers are listed in the 5′–3′ direction.

Gene	Forward Primer	Reverse Primer
*TFE3*	CCGTGTTCGTGCTGTTGGA	GCTCGTAGAAGCTGTCAGGAT
*VPS11*	CAAGCCTACAAACTACGGGTG	GAGTGCAGAGTGGATTGCCA
*VPS18*	CACTCGGGGTATGTGAATGCC	TCGGAAGGGGTGAAGTCAATG
*CTSD*	TGCTCAAGAACTACATGGACGC	CGAAGACGACTGTGAAGCACT
*CTSL*	CGTGACGCCAGTGAAGAATCA	CGCTCAGTGAGACAAGTTTCC
*Atg5*	AAAGATGTGCTTCGAGATGTGT	CACTTTGTCAGTTACCAACGTCA
*Atg16*	AACGCTGTGCAGTTCAGTCC	AGCTGCTAAGAGGTAAGATCCA
*LAMP1*	TCTCAGTGAACTACGACACCA	AGTGTATGTCCTCTTCCAAAAGC
*MCOLN1*	TTCGCCGTCGTCTCAAATACT	CTCTTCCCGGAATGTCACAGC
*FASN*	AAGGACCTGTCTAGGTTTGATGC	TGGCTTCATAGGTGACTTCCA
*ACC*	ATGTCTGGCTTGCACCTAGTA	CCCCAAAGCGAGTAACAAATTCT
*SCD1*	GCCCCTCTACTTGGAAGACGA	AAGTGATCCCATACAGGGCTC
*DGAT1*	TATTGCGGCCAATGTCTTTGC	CACTGGAGTGATAGACTCAACCA
*DGAT2*	ATTGCTGGCTCATCGCTGT	GGGAAAGTAGTCTCGAAAGTAGC
*PNPLA2*	ATGGTGGCATTTCAGACAACC	CGGACAGATGTCACTCTCGC
*LIPC*	ATCAAGTGCCCTTGGACAAAG	TGACAGCCCTGATTGGTTTCT
*PPARα*	TTCGCAATCCATCGGCGAG	CCACAGGATAAGTCACCGAGG
*PGC1α*	TCTGAGTCTGTATGGAGTGACAT	CCAAGTCGTTCACATCTAGTTCA
*CPT1α*	TCCAGTTGGCTTATCGTGGTG	TCCAGAGTCCGATTGATTTTTGC
*ACOX1*	ACTCGCAGCCAGCGTTATG	AGGGTCAGCGATGCCAAAC
*GAPDH*	CTGGGCTACACTGAGCACC	AAGTGGTCGTTGAGGGCAATG
